# Traveling itinerary problem in a scheduled multimodal transportation network for a fixed sequence of cities

**DOI:** 10.1371/journal.pone.0287604

**Published:** 2023-11-03

**Authors:** Rafaqat Ali, Hai Jiang, Lubos Buzna

**Affiliations:** 1 Department of Industrial Engineering, Tsinghua University, Beijing, P.R.China; 2 Department of Mathematical Methods and Operations Research, University of Zilina, Zilina, Slovakia; 3 Department of International Research Projects - ERADiate+, University of Zilina, Zilina, Slovakia; Southwest Jiaotong University, CHINA

## Abstract

Developing an efficient and economical journey plan in multimodal transportation networks is of significant and fast-growing importance, but it is still an annoying experience for a traveler. This paper aims to find the journey plan at a combined cross-border and inter-regional level when visiting a sequence of cities while utilizing several transport modes to reduce travel costs and planning time. We study a traveling itinerary problem in a scheduled multimodal transportation network with constraints on both arcs and nodes as a new extension of the shortest path problem. We formulate a 0-1 integer linear programming model for the traveling itinerary problem and develop an exact algorithm that finds a combined cross-border and inter-regional low costs journey plan. We present case studies based on real-world transport data to illustrate the usefulness and computational efficiency of the proposed approaches. We compare the results with the previously proposed approach to demonstrate the benefits of multimodal journeys. Finally, we compare the results with the solution obtained by the general-purpose 0-1 integer linear programming solver to evaluate the computational time.

## Introduction

With the rapid growth in human mobility, the transportation sector faces several challenges, such as planning and scheduling transportation services to accommodate passengers. An increased number of travelers brought more attention to the transportation sector, which leads to the development of transport infrastructure and services. Several transport services are developed for every destination with different time schedules to accommodate all passengers. The decision to focus on public transport is one of the keys to substantially reducing greenhouse emissions and increasing efficiency in the transport sector. The high level of tourism and travel activities worldwide motivate and justify initiatives leading to improvements in the transport sector, especially developing novel solutions algorithm for planning trips. There are numerous journey planners available today that help travelers. Google Maps, Citymapper, Moovit, and Transit are among the most popular. None of these journey planners supports planning of multi-day trips while controlling the stay time duration. Hence, there is a need for a comprehensive journey planner that addresses the need of travelers who plan multiple-day and multiple-cities itineraries with given stay times and other time constraints in multimodal transportation. Planning a multi-city cross-border and inter-regional travel itinerary in a multimodal transport system is a complex problem that requires proper planning, taking into account factors such as transport modes availability, journey times, time of departure, arrival, and cost. Such an algorithm can improve travelers’ travel experiences by reducing the time and effort needed to arrange a trip and by giving them access to more reliable and accurate information. Suppose a traveler wants to visit multiple cities in Germany, France, and Italy, and the sequence of cities they want to visit from their origin city (Munich) are Frankfurt, Paris, Marseilles, Milan, and Rome, with a three-day stay in each city. The objective is to minimize total travel costs and time while considering multimodal transport modes such as flights, buses, and trains and also considering the time constraints such as time of departure and arrival, earliest departure and the latest arrival, and stay time at each city. Having several possibilities of regular modes of transport and the number of connections to every destination with different schedules and costs, the complexity of planning a trip is increasingly high. Planning the trip individually and independently for every pair of cities in the sequence would create a confusing scenario. For example, when planning a trip from Munich to Rome while also visiting Frankfurt, Paris, Marseilles, Milan would require searching over all connections on how to get from Munich to Frankfurt. Then, when planning a trip from Frankfurt to Paris, it is needed to explore all connections where the departure time happens after all possible arrivals at Frankfurt and stay time. It is also likely that independent and individual trip planning for every pair of cities would cause the elimination of cost-effective links. For example, planning a trip from Munich to Frankfurt may lead to selecting a very cheap link. Still, it may not lead to a cheap solution when also considering the costs of getting from Frankfurt to Paris. Thus, breaking the planning problem into a sequence of pairwise searching problems creates a hard-to-handle scenario for travelers and may lead to sub-optimal results. Thus, there is a need for such algorithms that can be used by intelligent transportation systems that take all of these time constraints into account and recommend an optimized travel option for each leg of the trip in the multimodal transport network at the combined cross-border and inter-regional level.

### Related work

A Multimodal Transportation System (MTS) combines two or more modes of transport that can be used to reach the final destination. When planning an itinerary, a traveler may wish to reach their destination through multiple intermediate distant cities by multimodal transport within a given time frame. Planning itineraries becomes complex in multimodal transportation because of the plethora of possibilities for transport services, time schedules, and routes. The traveling Itinerary Problem (TIP) is a variant of the traveling salesman problem [1–3]. Many traveling salesman problem variants and generalizations have been studied extensively [4, 5]. Particularly relevant to this research is the shortest path problem [6–10] and the time-dependent shortest path problem with time windows also studied in [11–15].

Several approaches based on graph theory have been developed in the research community. Hedi et al. [16] proposed a transfer graph defined as a set of sub-graphs connected by a transfer point, which is a node that connects two distant components of the transfer graph, Ayed et al. [17] proposed a hybrid approach of transfer and relevant graph to solve the time dependent multimodal transportation network. In [18], the authors developed a hyper graph abstraction of a time-dependent multimodal transportation network that consists of two levels of abstraction, i.e., regions and transport modes. In [19], the authors modeled the network using a hierarchical structure. Dynamic segmentation and linear reference techniques are applied, and a three-layer transport system consisting of a physical layer (street network), logical layer (roadway networks), and application layer is constructed (bus, metro, BRT, etc), time-dependent graph [20, 21] and time expanded network [22]. Besides several algorithms, the Dijkstra algorithm [23] is still the most frequently used approach to compute the shortest path [17, 18, 24, 25]. For arcs having interval weights, [26] proposed a generalized Dijkstra algorithm to solve the shortest path problem. Enayattabar et al. [27] provided a solution to the shortest path problem using the Dijkstra algorithm, in which the arc is represented by interval-valued Pythagorean fuzzy numbers. At the same time, other algorithms like Tabu Search [28], Ant Colony algorithm [18], Artificial bee colony (ABC) algorithm [29], Branch-and-Cut [30], A* and ALT [31, 32], Fuzzy weighted Ant Colony optimization approach [33], hybrid of adaptive large neighborhood search with Tabu search [34], a hybrid of Genetic algorithm and Variable Neighborhood search (VNS) [24] and other heuristics and meta-heuristics [24, 30] have also been investigated.

Ref. [2] proposed a traveling itinerary problem to find the cheapest way how to visit multiple cities within a single mode of transportation. The author decomposed the problem into macroscopic and microscopic tours. An implicit enumeration algorithm was used to find the optimal combination of tours between the origin and destination city. A dynamic form of route planning was proposed by [31] to find the optimal path from a source to a destination based on the shortest path problem algorithm over a time-dependent multimodal graph. A hybrid of the Genetic algorithm and the Variable Neighborhood Search approach was proposed by [24] for route planning in a multimodal network. The hybrid approach gives better performance in terms of the quality of the final solution in comparison with the Genetic algorithm. Ref. [35] addressed a Profitable Tour Problem based on mixed-integer programming to elaborate travel itineraries to visit tourist attractions by considering the visitor profile and solved it using the exact branch and cut algorithm and heuristic Tabu search. Sori et al. [36] proposed fuzzy inference, that, considering cost, time, and risk factors, finds the optimal solution and the desirable path between origin and destinations. Ref. [37] studied the complexity of shortest paths in time-dependent graphs in which the costs of links vary as a function of time. Ref. [1] studied the time-dependent asymmetric traveling salesman problem with time windows with a constant travel time along with the links. Ref. [32] proposed a time-dependent shortest path problem with time windows intending to minimize the total arrival time at a destination in which costs along the links are also constant. Ref. [15] studied the *K*− shortest path problem in a time-scheduled network with constraints on links and proposed constant traverse costs along the links for different departure times. However, the real-world transportation network is more complex. The costs to traverse a link varies with different time schedules and modes of transportation.

New Mobility Services (NMS) include Mobility as a Service (MaaS) that provides users the option of choosing multimodal mode alternative [38, 39]. Integration of mobility services, enables offering a combination of different modes of transport for multiple cities with stay time to visit with a single interface. MaaS is still under development, but it could change the way we conceive transport and travel. The traveling itinerary problem in multimodal transportation networks is of significant and fast-growing importance. The aim is to find the route between origin and destination while utilizing multiple transport modes [19]. Different modifications of the time-dependent shortest path problems have been studied in the literature. Still, none of them addressed the problem for a given sequence of cities except [3], where the authors have studied the pre-determined travel plan in a single mode of transportation (flights). The authors decomposed the network into sub-networks based on time steps over the finite time horizon and searched for an optimal travel plan in a deterministic environment.

This paper studies the traveling itinerary problem for a fixed sequence of cities in multimodal transportation networks with time windows and other time constraints. The proposed approach is based on a directed scheduled multimodal transportation network (SMTN), constructed by integrating different modes of public transport in the sequence of cities. Next, we apply a network pruning algorithm to eliminate irrelevant transport connections. We developed an exact algorithm to find a low-cost journey plan in the SMTN under time constraints. Finally, to demonstrate the effectiveness of the proposed algorithm, we compare the results with solutions obtained by the general-purpose 0-1 integer programming solver. To the best of our knowledge, no study has previously addressed this problem in the literature.

### Contribution

This paper is motivated by the traveling itinerary problem found in the domain of public transport. In particular, we address combined inter-regional and cross-border journey planning. A sequence of cities is considered with multiple days to stay in each city within a planning time horizon and other time constraints while utilizing multimodal transportation. An optimal journey plan is searched for in a deterministic environment. Nowadays, several solutions exist to solve conventional route planning in transportation systems, such as Google, Bing, and Baidu Maps. Most such routing tools support more than one transport mode. Still, they do not support journey planning for multiple intermediate cities with multiple days to stay at each city in a multimodal transportation network. Developing an efficient and economical journey plan is still an annoying experience for a traveler. Although journey planners operated by transport service providers can provide some predefined itineraries, they are not tailored to a specific traveler. These journey planners are also limited to national or regional-level journey planning and do not support cross-border and inter-regional trips. Moreover, most journey planners only consider a single day’s trip, while in real cases, most travelers wish to schedule an *n*− days itinerary. This deficiency can also be viewed against huge cross-border multimodal travel demand within the European Union (EU), leading to about one hundred million cross-border trips every year by EU residents and several hundred million trips by international tourists [40]. In this paper, we design a more general itinerary planning service to address the above limitations.

The contributions of this paper are as follows: (i) First, we model our traveling itinerary problem as a Scheduled Multimodal Transportation Network (SMTN) through a fixed sequence of cities. Taking advantage of the particular structure of this model, we proposed an exact approach that allows us to solve problems of realistic size to optimality. (ii) We propose an approach that satisfies the unprecedented demand for intelligent and sustainable tourism-driven advances in big data. Our work can form the foundation for extensive models and support systems for decision support that will emerge as functional websites or mobile applications to plan regional or cross-border traveling itineraries in multimodal transportation. (iii) We bridge the gap between urgent travel demand and supply at the inter-regional and cross-border levels. Our solution approach shows computational effectiveness when applied to inter-regional and cross-border itinerary planning situations.

## Scheduled multimodal transportation network

The traveling itinerary problem consists of finding the best journey plan (itinerary) for a pre-determined sequence of cities based on the traveler’s preferences. In our approach, we integrate multiple transport modes (i.e., bus, train, flight, etc.) into a single network to find a cost-effective journey plan under time constraints.

Suppose a traveler plans to visit cities *V* = {1, 2, …, *n*} in a fixed order given by the node indexes, where 1 is the origin, and *n* is the destination city. The time constraints are given by the time horizon (defined by the start time $\bar{T}$ and the end time $\underline{T}$ of the trip), the earliest time of day of the departure $\hat{d}$, the latest time of day of the arrival $\hat{a}$ (both $\hat{d}$ and $\hat{a}$ are the same for all cities), maximum allowed traverse time *δ* along each link, and stay time at each city *s*_*i*_. Stay time is the length of period a traveler wants to spend in a city before visiting the next city. There is no stay time associated with the origin and destination nodes. Origin and destination cities may be the same. In that case, we consider them as two different cities.

**Fig 1:**
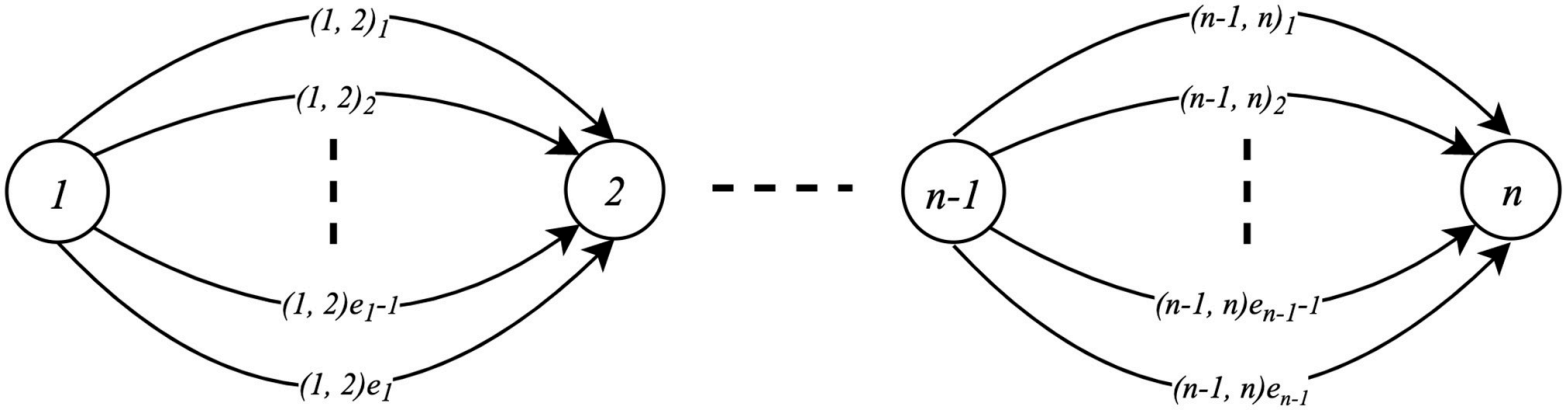
The graph representing the scheduled multimodal transportation network.

To compute the cheapest multimodal path between origin and destination, we model the scheduled multimodal transportation network by a graph *G* = (*V*, *A*), as shown in Fig 1. The idea behind the integration of transport connections into one network is to accommodate all the possible transport connections of different transport modes leaving and entering a city. Each city *i* ∈ *V* has *e*_*i*_ outgoing transport connections, i.e., $A=\{{\left(1,2\right)}_{1},\ldots ,{\left(1,2\right)}_{e_{1}},\ldots ,{\left(n-1,n\right)}_{1},\ldots ,{\left(n-1,n\right)}_{e_{n-1}}\}$. Each city *i* and transport connection *k* = 1, …, *e*_*i*_ are associated with a tuple [(*d*_(*i*,*k*)_, *a*_(*i*,*k*)_, *c*_(*i*,*k*)_)], where *c*_(*i*,*k*)_ is the monetary costs associated with the use of *k*-th transport connections departing from city *i*, *d*_(*i*,*k*)_ is its departure time, and *a*_(*i*,*k*)_ is the arrival time to the next city *i* + 1.

Based on [2] we formulate a 0-1 integer linear programming model for the traveling itinerary problem. Let *x*_(*i*,*k*)_ be a binary decision variable that takes the value one if and only if *k*^*th*^ outgoing alternative transport service from city *i* is used to get to the next city *i* + 1 in the sequence and zero otherwise. The mathematical model takes the form (1)–(10).

The objective function (1) of the traveling itinerary problem is to find the cheapest travel plan for a trip. If a traveler is searching for the quickest route rather than the cheapest route, the objective function can be written as $\sum_{i=1}^{n-1}\sum_{k=1}^{e_{i}}\tau_{\left(i,k\right)}x_{\left(i,k\right)}$ by replacing the cost (*c*_(*i*,*k*)_) with the duration of the connection (*τ*_(*i*,*k*)_). The constraints (2–10) ensure the feasibility of the traveling itinerary. First, the departure time from the origin city must take place later than the start time of trip $\bar{T}$, as it is assured by (2). Constraint (3) ensures that the arrival time at the destination city must be less or equal to the end time of the trip $\underline{T}$. As there are *k* alternative transport services from city *i* to the next city in the sequence, to ensure that exactly one alternative transport service is selected, we add degree constraints (4). Once a traveler arrives at an intermediate city *i*, stays there for at least *s*_*i*_ time units, as assured by constraints (5). Constraints (6) and (7) ensure that the total waiting time (i.e., the duration of time after the stay time till the arrival to the next city, which constitutes an unproductive time between two consequent stays) is not more than a given threshold *δ*.

We assume that a traveler prefers to take a transport connection from each city after a specific time of day called the earliest departure time $\hat{d}$ and to arrive at each city before a specific time of day called the latest arrival time $\hat{a}$. For example, a traveler may want to depart from each city after 10:00 a.m. and arrive before 10:00 p.m. Depending on the existence of such a preference, time constraints (8) and (9) are included, where *t*[*d*_(*i*,*k*)_] and *t*[*a*_(*i*,*k*)_] are the time segment of departure and arrival respectively. Finally, integrality constraints (10) require that the decision variables *x*_(*i*,*k*)_ are binary for all *i* ∈ *V* and *k* = 1, …, *e*_*i*_.

$$\begin{array}{cc} & \text{Minimize}\;\sum_{i=1}^{n-1}\sum_{k=1}^{e_{i}}c_{\left(i,k\right)}x_{\left(i,k\right)} \end{array}$$ (1)

$$\begin{array}{cc} & \text{subject}\;\text{to}\\ & \sum_{k=1}^{e_{1}}d_{\left(1,k\right)}x_{\left(1,k\right)}\geq \bar{T}, \end{array}$$ (2)

$$\begin{array}{cc} & \sum_{k=1}^{e_{n-1}}a_{\left(n-1,k\right)}x_{\left(n-1,k\right)}\leq \underline{T}, \end{array}$$ (3)

$$\begin{array}{cccc} & \sum_{k=1}^{e_{i}}x_{\left(i,k\right)}=1 & & \forall i\in V, \end{array}$$ (4)

$$\begin{array}{cccc} & \sum_{k=1}^{e_{i}}d_{\left(i,k\right)}x_{\left(i,k\right)}\geq \sum_{k=1}^{e_{i-1}}a_{\left(i-1,k\right)}x_{\left(i-1,k\right)}+s_{i} & & \forall i\in V-\{1\}, \end{array}$$ (5)

$$\begin{array}{cccc} & \sum_{k=1}^{e_{i}}a_{\left(i,k\right)}x_{\left(i,k\right)}\leq \sum_{k=1}^{e_{i-1}}a_{\left(i-1,k\right)}x_{\left(i-1,k\right)}+s_{i}+\delta & & \forall i\in V-\{1\}, \end{array}$$ (6)

$$\begin{array}{cc} & \sum_{k=1}^{e_{1}}a_{\left(1,k\right)}x_{\left(1,k\right)}\leq \bar{T}+\delta , \end{array}$$ (7)

$$\begin{array}{cccc} & \sum_{k=1}^{e_{i}}t\left[d_{\left(i,k\right)}\right]x_{\left(i,k\right)}\geq \hat{d} & & \forall i\in V, \end{array}$$ (8)

$$\begin{array}{cccc} & \sum_{k=1}^{e_{i}}t\left[a_{\left(i,k\right)}\right]x_{\left(i,k\right)}\leq \hat{a} & & \forall i\in V, \end{array}$$ (9)

$$\begin{array}{cccc} & x_{\left(i,k\right)}\in \left\{0,1\right\} & & \forall i\in V,k=1,2,\ldots ,e_{i}. \end{array}$$ (10)

## Solution algorithms

First, we apply the Network pruning algorithm to amend the scheduled multimodal transportation network *G* considering the constraints (2), (3), (8) and (9). Second, the journey plan is calculated by the Exact algorithm.

### Network pruning algorithm

The irrelevant transport connections that do not obey some of the constraints are eliminated from the graph representing the scheduled multimodal transportation network by the Network pruning algorithm. The Network pruning algorithm is divided into two parts. First, the irrelevant transport connections are removed based on the start of trip $\bar{T}$ and end of trip $\underline{T}$. Second, transport connections are eliminated based on the earliest acceptable time of day of a departure $\left(\hat{d}\right)$ and the latest time of day of an arrival $\left(\hat{a}\right)$. Below, we show the pseudo-code of the Network pruning algorithm.

**Algorithm 1:** Network pruning algorithm.

**Input**: G, $\bar{T}$, $\underline{T}$, $\hat{d}$, $\hat{a}$

**Output**: G

**1 for** *i = 1 to n* − 1 **do**

**2  for** *k = 1 to e*_*i*_ **do**

    // Part I: eliminate transport connections outside the trip time window

**3   if** *d*_(*i*,*k*)_ ≤ $\bar{T}$ **then**

**4**    remove (*i*, *i* + 1)_*k*_ from *G*

**5**   **end**

**6**   **if** *a*_(*i*,*k*)_ ≥ $\underline{T}$ **then**

**7**    remove (*i*, *i* + 1)_*k*_ from *G*

**8**    **end**

    // Part II: eliminate transport connections with too early departure or too late arrival

**9**   **if** $\hat{d}$, $\hat{a}$ *are given* **then**

**10**    **if** *t*[*d*_(*i*,*k*)_] ≤ $\hat{d}$ **then**

**11**     remove (*i*, *i* + 1)_*k*_ from *G*

**12**    **end**

**13**    **if** *t*[*a*_(*i*,*k*)_] ≥ $\hat{a}$ **then**

**14**     remove (*i*, *i* + 1)_*k*_ from *G*

**15**    **end**

**16**   **end**

**17**  **end**

**18** **end**

### Exact algorithm

The problem (1–10) can be interpreted as a problem to find the cheapest path in the SMTN that originates at city 1 and terminates at city *n* and obeys all constraints (2—10). To find such a path, we propose the Exact algorithm 1. The costs are additive, and hence to find the optimal path, the dynamic programming [41] can be applied. With each arc (*i*, *i* + 1)_*k*_ in the SMTN, we associate the value *ϕ*_(*i*,*j*)_, that represents the costs associated with the optimal path that is initiated at the origin city and terminates at city *i* + 1 and utilizes arc *j* exiting city *i*. To keep track of optimal paths, we define variables *P*_(*i*,*j*)_ that contains the index of the arc exiting city *i* − 1, that precedes arc *j* exiting city *i* in the optimal path that is initiated at the origin city and terminates at city *i* + 1.

First, all values *ϕ*_(*i*,*k*)_ are initialized. The computation starts from the city 1. Therefore, for the city 1, values *ϕ*_(1,*k*)_ are set to *c*_(1,*k*)_ for all *k* = 1, …, *e*_1_ where constraint (7) is satisfied and for all other cities and all others arcs to value ∞. Furthermore, cities are recursively processed in the order of visits, and for each city *i* = 2, …, *n*−1 and for each arc exiting the city *i*, the value of *ϕ*_(*i*,*j*)_ and the corresponding value *P*_(*i*,*j*)_ are established. Knowing the values *ϕ*_(*i*−1,*k*)_ for all *k* = 1, …, *e*_*i*−1_, the value *ϕ*_(*i*,*j*)_ is set to the minimum sum *ϕ*_(*i*−1, *k*)_ + *c*_*i*,*j*_ over all feasible *k*. The value of *k* which minimizes the sum is stored in *P*_(*i*,*j*)_. This way the costs of the optimal path utilizing the connection *e*_*i*,*j*_ from the city 1 to the city *i* are calculated. After calculating the values of *ϕ*_(*n*−1,*k*)_ for *k* = 1, …, *e*_*n*−1_, costs associated with the optimal path that is connecting cities 1 and *n* are determined as $\min_{k=1,\ldots ,e_{n-1}}\phi_{\left(n-1,k\right)}$. The optimal path can be found by backtracking utilizing values recorded in *P*_(*i*,*j*)_.

**Algorithm 2:** Exact algorithm.

**Input**: (G, *s_i_*, *δ*)

**Output**: P

//Initialization

**1 for** *i* = 1 to *n* − 1 **do**

**2**  **for** *k* = 1 to *e*_*i*_ **do**

**3**   **if** *(i == 1) and* ($a_{\left(i,k\right)}\leq \bar{T}+\delta$) **then**

**4**    *ϕ*_(*i*,*k*)_ ← *c*_(*i*,*k*)_

**5**   **end**

**6**   **else**

**7**    *ϕ*_(*i*,*k*)_ ← ∞

**8**   **end**

**9**  **end**

**10** **end**

**11** **for** *i = 2 to n* − 1 **do**

**12**  **for** *j = 1 to e*_*i*_ **do**

**13**   **for** *k = 1 to e*_*i*−1_ **do**

**14**    *new*_*costs* ← *ϕ*_(*i*−1,*k*)_ + *c*_(*i*,*j*)_

**15**    **if** *new*_*costs* < *ϕ*_(*i*,*j*)_ **then**

**16**     **if** *d*_(*i*,*j*)_ ≥ *a*_(*i*−1,*k*)_ + *s*_*i*_ **then**

**17**      **if** *a*_(*i*,*j*)_ ≤ *a*_(*i*−1,*k*)_ + *s*_*i*_ + *δ* **then**

**18**       *ϕ*_(*i*,*j*)_ ← *new*_*cost*

**19**       *P*_(*i*,*j*)_ ← *k*

**20**      **end**

**21**     **end**

**22**    **end**

**23**   **end**

**24**  **end**

**25** **end**

The optimality of the solution is implied by the Bellman’s optimality mechanism of the dynamic programming [41], and it can be simply proven by induction. According to this principle, an optimal solution to a problem includes an optimal solution to any related sub-problem. The proposed algorithm breaks down the problem into smaller sub-problems of finding an optimal itinerary for a shorter sub-sequences of cities. The algorithm stores the solution of sub-problems in the *ϕ*_(*i*,*k*)_. At each iteration, the algorithm examines all the possible connections between last city in the already optimized sub-problem and the next city. It is straightforward to see that values *ϕ*_(1,*k*)_ are set in the optimal way. Assuming that values *ϕ*_(*i*−1,*k*)_ are optimal, setting the values *ϕ*_(*i*,*j*)_ to the minimum value of *ϕ*_(*i*−1,*k*)_ + *c*_*i*,*j*_ over all feasible *k* is optimal, as the city *i* − 1 cannot be bypassed and there is no cheaper way how to reach city *i* + 1 while using arc *j* existing city *i*. The feasibility of the solution results from the data pruning and also from the used conditions. Application of the Network pruning algorithm 1 ensures that constraints (2), (3), (8) and (9) are obeyed. The constraints (4) and (10) are satisfied by definition as *P*_(*i*,*j*)_ maintains information about one arc index only. Constraints (5) are satisfied thanks to the condition on line 16 and similarly, constraints (6) are ensured by the condition on line 17. Finally, constraints (7) are enforced by the condition on the line 3. Thus, solutions encoded by variables *ϕ*_(*i*,*j*)_ and *P*_(*i*,*j*)_ obey all constraints (2)- (10) and are therefore feasible.

It is possible that the exact algorithm will not find a solution. For example, if all connections between a city pair are infeasible, the algorithm will return an incomplete itinerary up to the first city in the pair.

## Results and discussions

We present two data sets created based on real-world data from flight, train, and bus timetables to illustrate the feasibility and computational efficiency of the Exact algorithm. Furthermore, we present two illustrative examples and results of computational analysis. The proposed network model and algorithms were coded in Python 3.6. The optimization solver CPLEX 12.8 was used to implement and solve the model (1)-(10) while limiting the computational time to 3600 seconds. The experiments were run on a 2.6 GHz Intel Core i5 Mac-Book Pro with 16 GB of RAM running OS 10.14.6.

**Table 1:** Numbers of transport connections by transport mode in the European cities dataset.

City Pair	Buses	Trains	Flights
London—Paris	317	203	826
London—Berlin	145	58	923
London—Warsaw	12	0	613
London—Amsterdam	110	119	798
Paris—London	316	190	901
Paris—Berlin	361	882	746
Paris—Warsaw	46	0	1046
Paris—Amsterdam	389	330	538
Berlin—London	195	77	1007
Berlin—Paris	396	835	692
Berlin—Warsaw	255	154	758
Berlin—Amsterdam	392	380	558
Warsaw—London	2	0	559
Warsaw—Paris	45	0	415
Warsaw—Berlin	438	206	720
Warsaw—Amsterdam	81	0	314
Amsterdam—London	154	209	751
Amsterdam—Paris	362	274	562
Amsterdam—Berlin	415	391	438
Amsterdam—Warsaw	93	0	429

### Data

First, we collected the data for five European cities, London, Paris, Berlin, Warsaw, and Amsterdam, from publicly available sources. Flight data were obtained from the travel agency Trip [42], and train and bus data were collected from Omio journey planner website [43]. Second, we assembled a data set concerning five Chinese cities, Beijing, Chongqing, Changchun, Qingdao, and Shanghai, where the data for both flights and high-speed trains were taken from [2].

The raw data, available at https://github.com/ralikk/Transportation, contains links between each pair of cities, including the transport details, origin, destination, departure time, arrival time, transfers (stopover), and cost. Currently, exchanges or stopovers are considered a part of input data. Thus, input data already include indirect connections that pass through other cities where a traveler must change the mean of transport. A generalization of the proposed approach that incorporates exchanges into the search for travel connections is left for future works. Tables 1 and 2 show the number of connections for each mode of transportation, for both data sets. The European cities data set contains connections from 2019-04-01 until 2019-04-13, while the Chinese data set contains connections from 2015-11-10 until 2015-11-20.

**Table 2:** Numbers of transport connections by transport mode in the Chinese cities dataset.

City Pair	High-speed Train	Flights
Beijing—Chongqing	22	343
Beijing—Changchun	33	324
Beijing—Qingdao	132	312
Beijing—Shanghai	418	360
Shanghai—Beijing	418	342
Shanghai—Changchun	44	657
Shanghai—Chongqing	44	654
Shanghai—Qingdao	44	562
Shanghai—Shenzhen	66	73
Chongqing—Beijing	22	339
Chongqing—Changchun	0	126
Chongqing—Qingdao	11	220
Chongqing—Shanghai	44	694
Chongqing—Shenzhen	11	94
Qingdao—Beijing	132	438
Qingdao—Changchun	33	197
Qingdao—Chongqing	11	281
Qingdao—Shanghai	44	597
Changchun—Beijing	44	399
Changchun—Chongqing	0	132
Changchun—Qingdao	33	186
Changchun—Shanghai	44	671

### Illustrative example 1

Let us assume a traveler living in London (origin and destination city) with a plan to visit the sequence of cities: London → Paris → Berlin → Warsaw → Amsterdam → London. The traveler prefers to take the economy class and has a preference to stay in each intermediate city, namely, 2 days in Paris, 3 days in Berlin, 2 days in Warsaw, and 2 days in Amsterdam. The traveler has a preference for the earliest departure 08:00, and the latest arrival time 22:30 for every city. The traveler wants to plan the entire itinerary in the multimodal transportation network at once, instead of searching for each connection and travel mode separately, to achieve the minimum travel costs, under time constraints given by the time horizon, the stay time at each city, the earliest departure, and the latest arrival time.

First, the SMTN was constructed, including all available transport connections. Then, by using the Network pruning algorithm, all irrelevant transport connections were eliminated. The Exact algorithm is used to find the cheapest path for the sequence of cities under time constraints. The traveling itinerary obtained by the Exact algorithm is shown in Table 3 with total costs USD 308.

**Table 3:** Traveling itinerary developed for Illustrative example 1.

City pair	Departure Time	Arrival Time	Stopover	Mode	Mode details	Cost
London → Paris	2019-04-01 08:00	17:00	Direct	Bus	Euroline France	$ 29
Paris → Berlin	2019-04-03 18:20	20:05	Direct	Flight	Easy Jet	$ 65
Berlin → Warsaw	2019-04-06 23:55	08:05(+1)	Direct	Bus	FlixBus	$ 24
Warsaw → Amsterdam	2019-04-09 10:00	14:15	1 Stop	Flight	SAS Airlines	$ 114
Amsterdam → London	2019-04-11 17:15	17:35	Direct	Flight	Easy Jet	$ 76

Taking into account the average number of daily flights, buses, and train connections, there are 102 for London→ Paris, 151 for Paris→ Berlin, 88 for Berlin→ Warsaw, 30 for Warsaw→ Amsterdam and 85 connections for Amsterdam→ London. Thus, in total, there are about 3.4 × 10^9^ potential itinerary planning strategies (including the infeasible). It would take an unmanageable time if a traveler had to find the optimal itinerary manually. In comparison, the Exact algorithm took 14.10 milliseconds to construct the SMTN and to find a traveling itinerary.

**Fig 2:**
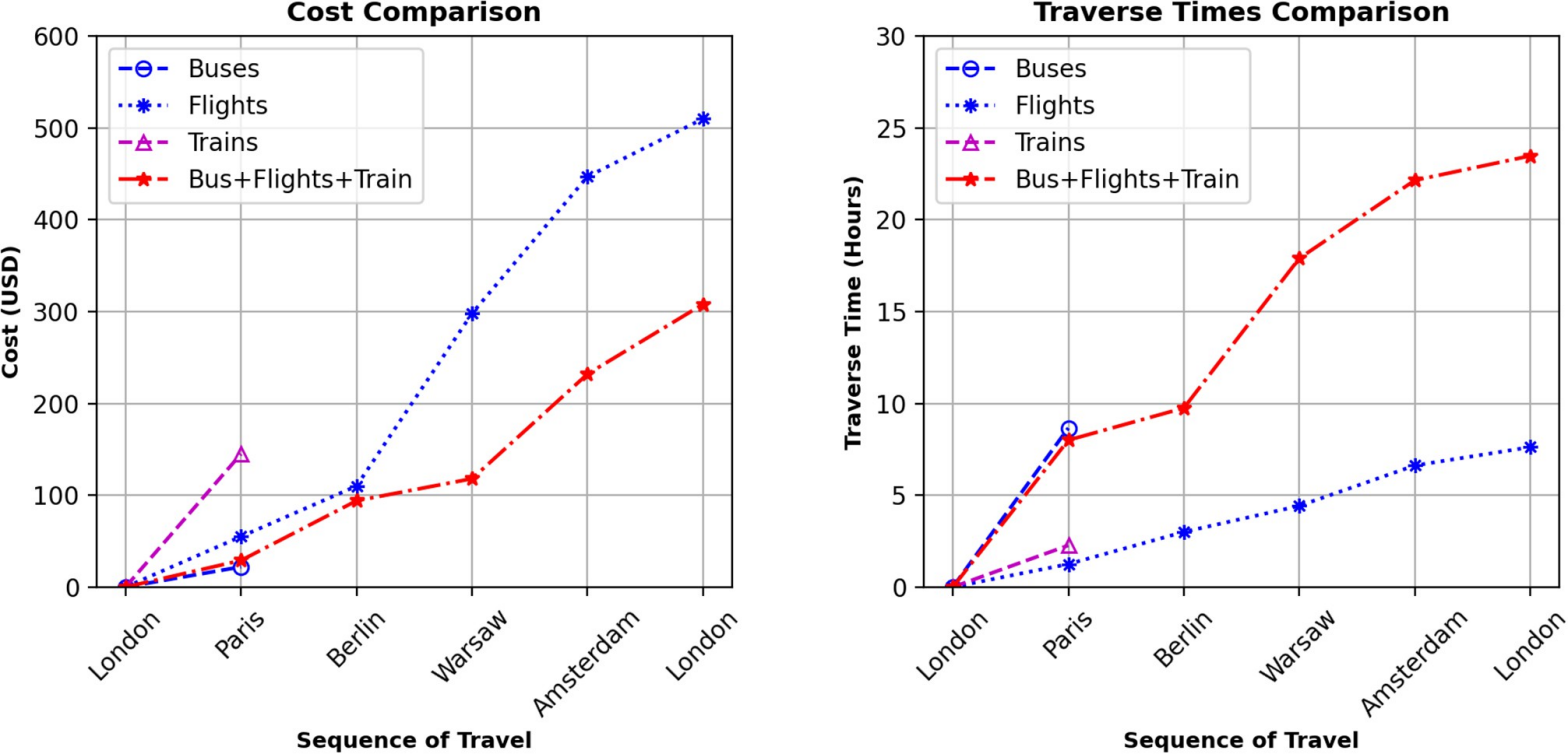
Illustrative example 1: Comparison of costs and traverse times for single and multiple modes of transport.

We developed single-mode and multimodal itineraries to demonstrate the effectiveness of traveling itineraries in the scheduled multimodal transportation network regarding travel costs and time. Fig 2 shows that the single-mode itineraries for buses and trains are not complete. This is because some pairs of cities in the sequence of travel do not have the desired transport connections, or there are no feasible connections available under time constraints. For example, in Fig 2 the train itinerary is incomplete and stops at Paris city because train connections are infeasible as traverse time exceeds the allowed waiting and traverse time (*δ* = 12*hr*). Thus, the results show that multimodal itineraries are more likely to be feasible and cost-effective than a single mode.

### Illustrative example 2

As Illustrative example 2, we consider the trip developed in [2] that is considering a sequence of Chinese cities and a single mode of transport (high-speed trains) with a total cost of 6369 RMB (Chinese Yuan). The sequence of cities with the stay time to visit are Beijing (origin city), Chongqing (1 day), Wuhan (1 day), Shenzhen (2 days), Shanghai (2 days), and Changchun (destination city). The time horizon for travel starts on 2015/11/10 at 08:00 and terminates on 2015/11/20 at 08:00, with the earliest departure time at 7:00 and the latest arrival time at 23:00, for each visited city. The Exact algorithm is used to find the cheapest path for the given sequence. The traveling itineraries developed by the approach developed in [2] and the Exact algorithm are compared in Table 4. The itinerary developed by the Exact algorithm is associated with costs of 3906.5 RMB (that is an optimal solution). The Exact algorithm took 5.10 milliseconds to construct SMTN and to find a traveling itinerary. Considering the multimodal approach proposed in this paper, for the same situation but a single-mode transport as presented in [2], the costs were reduced by 38.5%, as shown in Table 4.

**Table 4:** Traveling itinerary developed for Illustrative example 2.

City pair	Departure Time	Arrival Time	Stopover	Mode	Mode details	Cost
**Solution provided by approach developed in [2]**						
Beijing → Chongqing	2015-11-10 08:30	20:46	Direct	Train	G307	2383.0
Chongqing → Wuhan	2015-11-12 08:14	14:53	Direct	Train	G310	743.0
Wuhan → Shenzhen	2015-11-14 14:27	19:11	Direct	Train	G73	998.0
Shenzhen → Shanghai	2015-11-16 07:00	19:01	Direct	Train	D2282	593.5
Shanghai → Changchun	2015-11-19 09:13	23:15	Direct	Train	G1258	1651.5
					Total Cost	6369.0 RMB
**Solution provided by the Exact algorithm**						
Beijing → Chongqing	2015-11-10 22:00	00:55(+1)	Direct	Flight	Sichuan Air 3U8834	584.0
Chongqing → Wuhan	2015-11-12 08:14	14:53	Direct	Train	G310	743.0
Wuhan → Shenzhen	2015-11-13 15:33	20:53	Direct	Train	G131	1658.0
Shenzhen → Shanghai	2015-11-15 07:00	19:01	Direct	Train	D2282	593.5
Shanghai → Changchun	2015-11-18 07:50	10:40	Direct	Flight	Eastern Airline MU544	328.0
					Total Cost	3906.5 RMB

**Fig 3:**
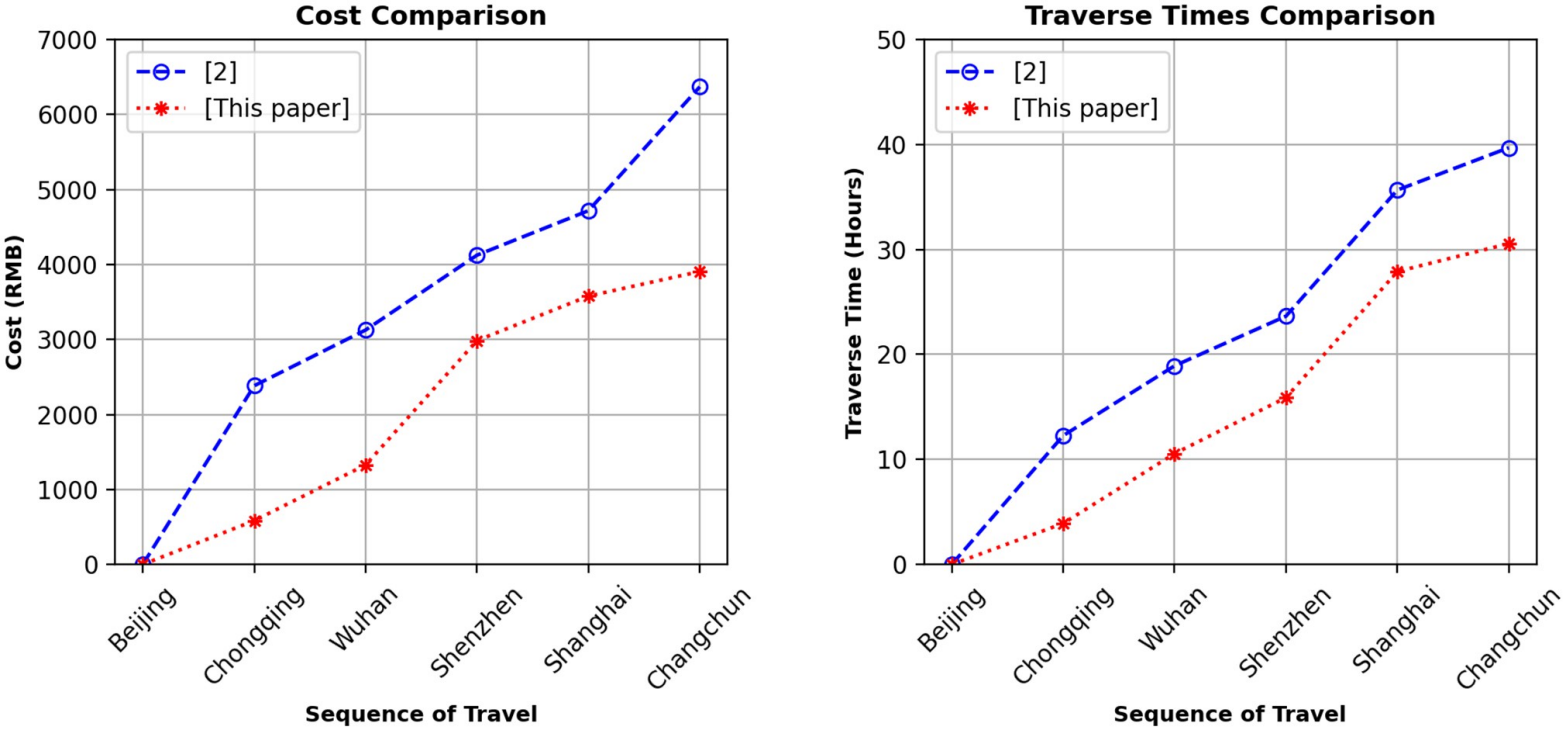
Illustrative example 2: Comparison of costs and traverse times developed by [2] and this paper.

Analogously to Fig 2, in Fig 3 we compare single mode (high-speed train) with multimodal (high-speed train and flights) itineraries obtained for Illustration example 2. The results again imply that the multimodal itineraries are complete and cost-effective as compared to single modes, highlighting the superiority of the proposed approach over the approach presented in [2].

**Table 5:** Comparison of results obtained by CPLEX, and Exact algorithm (run times are reported in milliseconds).

Number of Cities	SMTN Graph Development	Network Pruning Algorithm	Exact Algorithm		CPLEX	
			Solution Value	Time	Optimal Value	Time
**European cities:**						
3	1.35	0.0019	143	2.73	143	100
3	1.06	0.0012	139	2.17	139	180
3	1.02	0.001	46	2.33	46	60
3	1.07	0.0012	151	2.13	151	80
3	1.14	0.001	256	2.15	256	50
3	1.87	0.001	214	2.5	214	160
4	2.29	0.0019	66	4.49	66	260
4	2.13	0.0012	238	3.12	238	180
4	2.06	0.0012	177	6.82	177	190
4	2.18	0.001	203	3.25	203	180
4	2.09	0.0012	352	3.73	352	170
4	2.09	0.001	232	6.95	232	340
5	2.41	0.0021	186	6.84	186	430
5	2.29	0.0012	347	5.61	347	190
5	2.54	0.001	200	6.05	200	500
5	2.18	0.0021	253	5.32	253	570
5	2.26	0.0007	284	6.55	284	1410
5	2.27	0.001	147	5.84	147	390
6	2.52	0.0021	206	8.78	206	470
6	2.46	0.0021	287	9.27	287	460
6	2.64	0.0019	515	8.87	515	630
6	2.42	0.0007	256	6.74	256	570
6	2.41	0.0012	260	8.94	260	480
6	2.43	0.0019	281	7.95	281	370
**Chinese cities:**						
3	1.96	0.0007	1108	1.04	1108	10
3	1.87	0.001	897	1.01	897	10
3	1.93	0.0021	983	1.36	983	10
3	1.85	0.0021	658	2.25	658	20
3	1.95	0.0021	2114	1.93	2114	70
3	1.93	0.0012	1261	0.75	1261	10
4	2.17	0.001	1431	1.43	1431	40
4	2.11	0.0012	1272	1.41	1272	40
4	2.04	0.001	1099	1.85	1099	40
4	2.03	0.0012	914	2.56	914	120
4	2.28	0.001	1153	1.19	1153	60
4	2.04	0.001	1538	1.23	1538	50
5	2.34	0.0019	1580	2.55	1580	90
5	2.22	0.001	1694	3.92	1694	60
5	2.27	0.0012	1752	4.1	1752	50
5	2.2	0.001	1223	4.02	1223	100
5	2.21	0.001	1312	3.8	1312	110
5	2.21	0.001	1456	3.35	1456	80
6	2.39	0.0012	1733	6.17	1733	180
6	2.54	0.001	2132	4.36	2132	160
6	2.39	0.0012	1956	4.19	1956	110
6	2.38	0.0012	1729	5.05	1729	60
6	2.66	0.0019	1555	5.34	1555	80
6	2.41	0.0012	1862	5.77	1862	110

### Computational analysis

Computational experiments are performed for a number of different travel sequences of cities with different time horizons and preferred earliest departure and arrival times. For each case, we used six different sequences of the same length of cities and calculated the run time (sequence details are available at https://github.com/ralikk/Transportation). We performed the experiments with both data sets (Chinese and European) with different cities in the sequence and sequence lengths to illustrate the computational efficiency of the proposed algorithms. The SMTN Graph Development refers to the run time taken by building a scheduled multimodal transportation network that also include the data management, while columns 3 and 4(b) refer to the run time taken by algorithms 1, and 2, respectively. Table 5 illustrates that the computation run time for the European cities (as we have three modes of transportation) varies from 2.13 milliseconds to 9.27 milliseconds for the Exact algorithm, while for the Chinese cities (as we have two modes of transportation only) it varies from 0.75 milliseconds to 6.17 milliseconds. The run time goes up with increasing the number of cities in the sequence and modes of transportation.

The comparison of the performance of the Exact algorithm with the CPLEX is shown in Table 5. The solution obtained by the Exact algorithm is optimal, and hence we do not report the optimality gap. As expected, the CPLEX features a more extensive run-time in comparison with the Exact algorithm. The CPLEX run-time varies from 46 milliseconds to 515 milliseconds for the European cities data-set and from 658 milliseconds to 2132 milliseconds for the Chinese cities data set. The Exact algorithm run time varies from 3.20 milliseconds to 11.73 milliseconds for the European cities data-set and from 2.68 milliseconds to 8.18 milliseconds for the Chinese cities data set. Hence, the Exact algorithm provided an optimal solution with a much smaller run-time compared to the optimal solution provided by CPLEX.

The size of travel plans in computational experiments is limited to six cities. This size is greater than the majority of real-world trips. We can derive some expectations about the performance of the Exact algorithm on larger networks based on the structure of algorithms 1 and 2 and results shown in Table 5. The number of computations in the Network pruning algorithm grows linearly with the length of the sequence of cities and with the number of connections between cities. The number of computations in the Exact algorithm grows linearly with the length of the sequence of cities and quadratically with the number of connections between cities. Table 5 confirms that computational times grow mildly with the problem size. Hence, extending the number of cities should not lead to a considerable increase in the computational time, and the algorithm will remain practically applicable.

## Conclusions

In response to the growing need for more complex journey planners, this study aims to develop a time scheduling system for a multimodal transportation network, which helps travelers plan their multiple-day itineraries by reaching low traveling costs when visiting a sequence of cities with given stay times and other time constraints. Considering these requirements, we formulated a traveling itinerary problem as a generalization of the shortest path problem. Our approach provides an optimal itinerary for a fixed sequence of cities. We proposed integrating multiple transport modes to achieve a complete schedule for a given sequence of cities to visit. We used the Network pruning algorithm to confine the network according to the user preferences and applied the Exact algorithm to find an optimal itinerary. The proposed approach shows effectiveness in terms of computation time to plan the itinerary under time constraints. The proposed method saves travelers time when planning a trip and provides them with a well-defined itinerary. The development of more advanced journey planners can boost tourism and help close the gap between travel demand and supply.

### Limitations and future outlook

As the number of intermediate cities increases, the number of strategies for connection selection increases exponentially so that the computation time for itinerary optimization rises rapidly. Given the complexity caused by the increase in the number of intermediate cities, we may implement the proposed algorithms with a Map-Reduce computing framework and perform them on some distributed Cloud computing platforms, which could definitely report the optimal travel itineraries with significantly reduced computational time. In this study, exchange or stopover points are considered as data and can be included as a point of transfer in the multimodal transportation network. We do not take hotel expenses into consideration, which are an important portion of the travel plan costs. In our future research, we will consider the costs of staying (hotel reservation costs) at intermediate cities, develop alternative traveling itineraries for a set of cities instead of a fixed sequence, including time-scheduled intra-cities public transport, and design more sophisticated rules to identify irrelevant data. The travel distance and geographical conditions between different cities are different and optional travel modes may also be different. In this paper, the algorithm does not provide optional travel modes passengers can choose in different links. In the future, we will develop more intelligent and robust algorithms, where the passengers will have the option to choose travel mode in different links and also will have the option to choose the service type (business class, economic class, high-speed train, etc). We have only studied the deterministic approach. In the future, we will extend this work to ensure the reliability of planning inter-city travel in advance before departure.

## References

[1] Anna Arigliano, Gianpaolo Ghiani, Antonio Grieco. Time Dependent Traveling Salesman Problem with Time Windows:Properties and an Exact Algorithm. 2014

[2] Li X, Zhou J, Zhao X. Travel itinerary problem. Transportation Research Part B: Methodological. 2016;91:332–343. doi: 10.1016/j.trb.2016.05.013

[3] Bérubé JF, Potvin JY, Vaucher J. Time-dependent shortest paths through a fixed sequence of nodes: application to a travel planning problem. Computers & Operations Research. 2006;33(6):1838–1856. doi: 10.1016/j.cor.2004.11.021

[4] Yang HH, Chen YL. Finding K shortest looping paths with waiting time in a time–window network. Applied Mathematical Modelling. 2006;30(5):458–465. doi: 10.1016/j.apm.2005.05.005

[5] Glenn AM. Algorithms for the Shortest Path Problem with Time Windows and Shortest Path Reoptimization in Time-Dependent Networks. PhD thesis, Massachusetts Institute of Technology 2001.

[6] Sun J, Dong H, Kong Y, Fang Y. Solution to Shortest Path Problem Using a Connective Probe Machine. Mathematical Problems in Engineering. 2019;2019:1–8. doi: 10.1155/2019/9609302

[7] Alam MA, Faruq MO. Finding Shortest Path for Road Network Using Dijkstra’s Algorithm. Bangladesh Journal of Multidisciplinary Scientific Research. 2019;1(2):41–45. doi: 10.46281/bjmsr.v1i2.366

[8] Wang XZ. The Comparison of Three Algorithms in Shortest Path Issue. Journal of Physics: Conference Series. 2018;1087:022011.

[9] Kumar G, Bajaj RK, Gandotra N. Algorithm for Shortest Path Problem in a Network with Interval-valued Intuitionistic Trapezoidal Fuzzy Number. Procedia Computer Science. 2015;70:123–129. doi: 10.1016/j.procs.2015.10.056

[10] Ortega-Arranz H, Llanos DR, Gonzalez-Escribano A. The Shortest-Path Problem: Analysis and Comparison of Methods. Synthesis Lectures on Theoretical Computer Science. Cham: Springer International Publishing; 2015. Available from: https://link.springer.com/10.1007/978-3-031-02574-7.

[11] Jaballah R, Veenstra M, Coelho LC, Renaud J. The time-dependent shortest path and vehicle routing problem. INFOR: Information Systems and Operational Research. 2021;59(4):592–622.

[12] Omer J, Poss M. Time dependent shortest paths with discounted waits. Networks. 2019;74(3):287–301. doi: 10.1002/net.21885

[13] Di Puglia Pugliese L, Ferone D, Festa P, Guerriero F. Shortest path tour problem with time windows. European Journal of Operational Research. 2020;282(1):334–344. doi: 10.1016/j.ejor.2019.08.052

[14] Montero A, Méndez-Díaz I, Miranda-Bront JJ. An integer programming approach for the time-dependent traveling salesman problem with time windows. Computers & Operations Research. 2017;88:280–289. doi: 10.1016/j.cor.2017.06.026

[15] Jin W, Chen S, Jiang H. Finding the K shortest paths in a time-schedule network with constraints on arcs. Computers & Operations Research. 2013;40(12):2975–2982. doi: 10.1016/j.cor.2013.07.005

[16] Ayed H, Khadraoui D, Habbas Z, Bouvry P, Merche JF. Transfer Graph Approach for Multimodal Transport Problems. In: Le Thi HA, Bouvry P, Pham Dinh T, editors. Modelling, Computation and Optimization in Information Systems and Management Sciences. vol. 14. Berlin, Heidelberg: Springer Berlin Heidelberg; 2008. p. 538–547. Available from: http://link.springer.com/10.1007/978-3-540-87477-5_57.

[17] Ayed H, Galvez-Fernandez C, Habbas Z, Khadraoui D. Solving time-dependent multimodal transport problems using a transfer graph model. Computers & Industrial Engineering. 2011;61(2):391–401. doi: 10.1016/j.cie.2010.05.018

[18] Ayed H, Habbas Z, Khadraoui D, Galvez-Fernandez C. A parallel algorithm for solving time dependent multimodal transport problem. In: 2011 14th International IEEE Conference on Intelligent Transportation Systems (ITSC). Washington, DC, USA: IEEE; 2011. p. 722–727. Available from: http://ieeexplore.ieee.org/document/6082973/.

[19] Wang Xb, Zhang Gj, Hong Z, Guo Hf, Yu L. Modeling and Implementing Research of Multimodal Transportation Network. In: 2009 First International Conference on Information Science and Engineering. Nanjing, China: IEEE; 2009. p. 2100–2103. Available from: http://ieeexplore.ieee.org/document/5455613/.

[20] López D, Lozano A. Techniques in Multimodal Shortest Path in Public Transport Systems. Transportation Research Procedia. 2014;3:886–894. doi: 10.1016/j.trpro.2014.10.068

[21] Ziliaskopoulos A, Wardell W. An intermodal optimum path algorithm for multimodal networks with dynamic arc travel times and switching delays. European Journal of Operational Research. 2000;125(3):486–502. doi: 10.1016/S0377-2217(99)00388-4

[22] Fischer F, Helmberg C. Dynamic graph generation for the shortest path problem in time expanded networks. Mathematical Programming. 2014;143(1-2):257–297. doi: 10.1007/s10107-012-0610-3

[23] Dijkstra EW. A note on two problems in connexion with graphs. Numerische Mathematik. 1959;1:269–271. doi: 10.1007/BF01386390

[24] Dib O, Manier MA, Caminada A. Memetic Algorithm for Computing Shortest Paths in Multimodal Transportation Networks. Transportation Research Procedia. 2015;10:745–755. doi: 10.1016/j.trpro.2015.09.028

[25] Peng W, Hu X, Zhao F, Su J. A Fast Algorithm to Find All-Pairs Shortest Paths in Complex Networks. Procedia Computer Science. 2012;9:557–566. doi: 10.1016/j.procs.2012.04.060

[26] Ebrahimnejad A. An acceptability index based approach for solving shortest path problem on a network with interval weights. RAIRO—Operations Research. 2021;55:S1767–S1787. doi: 10.1051/ro/2020033

[27] Enayattabar M, Ebrahimnejad A, Motameni H. Dijkstra algorithm for shortest path problem under interval-valued Pythagorean fuzzy environment. Complex & Intelligent Systems. 2019;5(2):93–100. doi: 10.1007/s40747-018-0083-y

[28] Gmira M, Gendreau M, Lodi A, Potvin JY. Tabu search for the time-dependent vehicle routing problem with time windows on a road network. European Journal of Operational Research. 2021;288(1):129–140. doi: 10.1016/j.ejor.2020.05.041

[29] Ebrahimnejad A, Enayattabr M, Motameni H, Garg H. Modified artificial bee colony algorithm for solving mixed interval-valued fuzzy shortest path problem. Complex & Intelligent Systems. 2021;7(3):1527–1545. doi: 10.1007/s40747-021-00278-0

[30] Gavalas D, Konstantopoulos C, Mastakas K, Pantziou G, Vathis N. Heuristics for the time dependent team orienteering problem: Application to tourist route planning. Computers & Operations Research. 2015;62:36–50. doi: 10.1016/j.cor.2015.03.016

[31] Idri A, Oukarfi M, Boulmakoul A, Zeitouni K, Masri A. A new time-dependent shortest path algorithm for multimodal transportation network. Procedia Computer Science. 2017;109:692–697. doi: 10.1016/j.procs.2017.05.379

[32] El-Sherbeny NA. The Algorithm of the Time-Dependent Shortest Path Problem with Time Windows. Applied Mathematics. 2014;05(17):2764–2770. doi: 10.4236/am.2014.517264

[33] Di Caprio D, Ebrahimnejad A, Alrezaamiri H, Santos-Arteaga FJ. A novel ant colony algorithm for solving shortest path problems with fuzzy arc weights. Alexandria Engineering Journal. 2022;61(5):3403–3415. doi: 10.1016/j.aej.2021.08.058

[34] Pan B, Zhang Z, Lim A. A hybrid algorithm for time-dependent vehicle routing problem with time windows. Computers & Operations Research. 2021;128:105193. doi: 10.1016/j.cor.2020.105193

[35] da Silva AA, Morabito R, Pureza V. Optimization approaches to support the planning and analysis of travel itineraries. Expert Systems with Applications. 2018;112:321–330. doi: 10.1016/j.eswa.2018.06.045

[36] Abbaszadeh Sori A, Ebrahimnejad A, Motameni H. The fuzzy inference approach to solve multi-objective constrained shortest path problem. Journal of Intelligent & Fuzzy Systems. 2020;38(4):4711–4720. doi: 10.3233/JIFS-191413

[37] Foschini L, Hershberger J, Suri S. On the Complexity of Time-Dependent Shortest Paths. Algorithmica 2014; 68, 1075–1097. doi: 10.1007/s00453-012-9714-7

[38] Martinčević I, Brlek P, Domjan Kačarević N. Mobility as a Service (MaaS) as a Sustainability Concept for Tourist Destinations. Sustainability. 2022;14(12):7512. doi: 10.3390/su14127512

[39] Storme T, Casier C, Azadi H, Witlox F. Impact Assessments of New Mobility Services: A Critical Review. Sustainability. 2021;13(6):3074. doi: 10.3390/su13063074

[40] The Linking Danube Concept. Interreg Danube Transnational Programme;. Available from: https://www.interreg-danube.eu.

[41] Bellman R. The theory of dynamic programming. Bulletin of the American Mathematical Society. 1954;60(6):503–515. doi: 10.1090/S0002-9904-1954-09848-8

[42] Travel deals and promotions;. Available from: http://www.trip.com/.

[43] (goeuro) | omio;. Available from: http://www.omio.com/.

